# Multiple Patterns of Axonal Collateralization of Single Layer III Neurons of the Rat Presubiculum

**DOI:** 10.3389/fncir.2019.00045

**Published:** 2019-07-12

**Authors:** Yoshiko Honda, Takahiro Furuta

**Affiliations:** ^1^Department of Anatomy, School of Medicine, Tokyo Women’s Medical University, Tokyo, Japan; ^2^Department of Oral Anatomy and Neurobiology, Graduate School of Dentistry, Osaka University, Osaka, Japan

**Keywords:** single neuronal tracing, Sindbis viral vector, axonal arborization, morphology, postsubiculum, medial entorhinal cortex

## Abstract

The presubiculum plays a key role in processing and integrating spatial and head-directional information. Layer III neurons of the presubiculum provide strong projections to the superficial layers of the medial entorhinal cortex (MEC) in the rat. Our previous study revealed that the terminal distribution of efferents from layer III cells of the presubiculum was organized in a band-like fashion within the MEC, and the transverse axis of these zones ran parallel to the rhinal fissure. Identifying axonal branching patterns of layer III neurons of the presubiculum is important to further elucidate the functional roles of the presubiculum. In the present study, we visualized all axonal processes and terminal distributions of single presubicular layer III neurons in the rat, using *in vivo* injection of a viral vector expressing membrane-targeted palmitoylation site-attached green fluorescent protein (GFP). We found that layer III of the rat presubiculum comprised multiple types of neurons (*n* = 12) with characteristic patterns of axonal collateralization, including cortical projection neurons (*n* = 6) and several types of intrinsic connectional neurons (*n* = 6). Two of six cortical projection neurons provided two or three major axonal branches to the MEC and formed elaborate terminal arbors within the superficial layers of the MEC. The width and axis of the area of their terminal distribution resembled that of the band-like terminal field seen in our massive-scale observation. Two of the other four cortical projection neurons gave off axonal branches to the MEC and also to the subiculum, and each of the other two neurons sent axons to the subiculum or parasubiculum. Patterns of axonal arborization of six intrinsic connectional neurons were distinct from each other, with four neurons sending many axonal branches to both superficial and deep layers of the presubiculum and the other two neurons showing sparse axonal branches with terminations confined to layers III–V of the presubiculum. These data demonstrate that layer III of the rat presubiculum consists of multiple types of cortical projection neurons and interneurons, and also suggest that inputs from a single presubicular layer III neuron can directly affect a band-like zone of the MEC.

## Introduction

Connectivity among the hippocampal formation [dentate gyrus, cornu ammonis (CA), and subiculum], presubiculum (PreS) and entorhinal cortex (EC) are crucial for memory formation. Sensory information converges on EC and is transmitted to the hippocampal formation. Signals are processed in the internal circuit, propagated to the cortical and subcortical structures, then return to EC. Back projections from the hippocampal formation to EC include two pathways: one a direct back pathway (i.e., from CA1 and the subiculum to the deep layers of EC; Tamamaki and Nojyo, 1995); and the other an indirect pathway. This indirect back pathway mainly originates in the subiculum (Sub) and reaches EC *via* PreS or parasubiculum (ParS; Caballero-Bleda and Witter, 1994). We have studied the major projections from layer III of PreS to the superficial layers of the medial entorhinal cortex (MEC) and found that the presubicular projections terminated in a band-like zonal area of MEC (Honda and Ishizuka, 2004). The transverse axes of these zones were disposed parallel to the rhinal fissure and their longitudinal axes were perpendicular to the boundary between MEC and the lateral entorhinal cortex (LEC). This finding raises the question of whether the terminal arborizations of each single entorhinal projection neuron in layer III of PreS constitutes such a band-like zone. However, little is known about the axonal branching patterns of each presubicular neuron (Honda et al., 2011). To address this, we visualized all axonal processes of single presubicular neurons in layer III, using *in vivo* injection of a virally expressed membrane-targeted palmitoylation site-attached green fluorescent protein (palGFP; Furuta et al., 2001). This vector is useful as a highly sensitive anterograde tracer for tracing long, finely arborized axonal branches (Kuramoto et al., 2009; Matsuda et al., 2009). Axonal tracing through many serial sections enabled identification of multiple types of single layer III neurons in rat PreS based on axonal morphology.

## Materials and Methods

The present experiments were approved by the Animal Care and Use Committee of Tokyo Women’s Medical University, and all conformed to the Guidelines for the Care and Use of Laboratory Animals (National Institutes of Health, USA). We used eight adult male Wistar rats (280–305 g body weight; Clea Japan, Tokyo, Japan), with every effort made to minimize the number of animals used and the pain and distress of animals.

### Constitution of Recombinant Sindbis Virus

The DNA construct containing the sequences for GFP tagged with a membrane-targeting palmitoylation site was inserted into the PmaCI site of pSinRep5 (Invitrogen, Carlsbad, CA, USA) as fully described by Furuta et al. (2001). The recombinant Sindbis virus, which was produced with the pSinRep5 containing the construct, was replication deficient and designed so that infected cells would express palGFP under the control of a powerful sub-genomic promoter of the virus.

### Injection of Viral Vector

Rats were initially sedated with 5% isoflurane and a surgical level of anesthesia was maintained by intramuscular injection of a mixture of ketamine (60 mg/kg body weight) and xylazine (20 mg/kg body weight). Each animal was placed in a stereotaxic frame, and a hole was drilled in the skull at coordinates derived from the atlas of (Paxinos and Watson, 1998; Table 1). Between 2,000 and 4,000 infectious units (IU) of palGFP-expressing Sindbis virus vector (Furuta et al., 2001) in 0.5–1.0 μl of 5 mM phosphate-buffered saline (PBS; pH 7.4) containing 0.5%–2.0% bovine serum albumin was pressure-injected stereotactically into part of PreS through a glass micropipette attached to a Picospritzer II (General Valve, Fairfield, NJ, USA). All injections were performed unilaterally (left side).

**Table 1:** The location of cell bodies and number of main axon collaterals to the termination areas or layers from each layer III neuron of PreS.

		Location of cell body				Number of labeled axonal arbors											
Cell No. (Rat No.)	†	sept-temp	prox-dist	depth	Neuron Type	PreS (layer)						Sub	MEC		ParS (sup/ deep)	cc, dhc	fim
						II	III			V	VI		I–III	V–VI			
							sup	m	deep								
#5 (41)	a	sept	m	sup	np	2	6	4	0	1	2	1	0	0	0	0	0
#8 (81)	a	m-sept	m	m	p	0	4	12	4	0	1	0	0	0	0	0	0
#4 (41)	a	m-sept	prox	m	p	0	0	2	0	0	4	8	0	1	0	1	0
#11 (72)	a	m-sept	dist	m	p	0	0	6	2	0	0	0	0	0	0	0	0
#9 (36)	c	m	prox	sup	np	2	6	5	5	1	0	0	0	0	0	0	0
#1 (18)	a	m	m	m	p	0	0	0	0	0	2	0	*	0	0	2	0
#3 (41)	a	m	m	deep	p	7	1	1	4	0	21	3	5	1	0	2	1
#10 (27)	b	m	dist	sup	np	3	12	0	4	0	0	0	0	0	0	0	0
#7 (19)	a	m	dist	m	np	3	2	3	19	6	4	0	0	0	0	0	0
#6 (19)	a	m	dist	m	p	0	0	1	1	*	1	0	0	0	17 (9/8)	0	0
#12 (81)	b	m	dist	deep	np	0	0	0	3	2	0	0	0	0	0	0	0
#2 (28)	c	m-temp	m	sup	p	0	3	2	0	0	1	0	*	0	0	0	0

*Making terminal plexus, m, middle; np, non-pyramidal; p, pyramidal; sup, superficial. ^†^Coordination of the injection site: a = AP-7.3, ML-3.2, D+3.4, b = AP-7.2, ML-4.1, D+4.8, c = AP-7.32, ML-4.1, D+4.8. The density patterns of shading indicate the approximate quantity of axonal arbors.

### Fixation and Cutting

**Figure 1:**
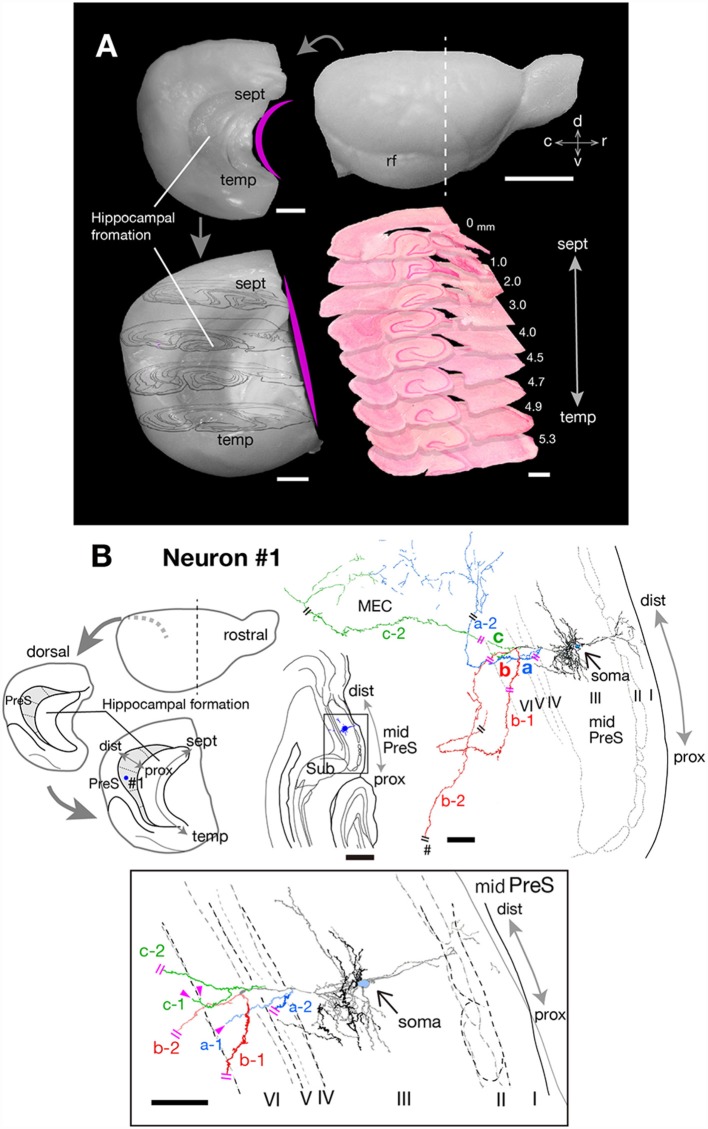
**(A)** Explanatory diagrams of the preparation of extended hippocampus and the cutting plane perpendicular to the longitudinal (septotemporal) axis of the hippocampus. Upper right is the right-side view of a rat brain. The white dotted line indicates the first cutting-plane line, which cut-off the frontal brain block. Upper left is a curved left hemisphere, which is removed from the diencephalon. Lower left is a flattened left hemisphere, in which the longitudinal axis of the hippocampal formation is approximately linear and the drawings of four cutting planes are superimposed on it. Lower right is the superposition of nine transverse sections of the flattened left hemisphere, perpendicular to the longitudinal axis of the hippocampus. **(B)** Schematic diagrams of the extended hippocampus as shown in **(A)**. Shaded regions represent PreS and the septotemporal parcellation (see also Figure 6) is indicated by small dotted lines. The localization of cell body of neuron #1 is represented as a blue-colored circle. A drawing of the section which includes the cell body and several dendrites (upper middle) and camera lucida reconstruction of the axonal morphology (upper right) of neuron #1. The cell body is painted over in blue. Three main axonal branches a, b and c and their collaterals are represented by blue, red and green, respectively. Dendrites and the main axon that comes directly out of the cell body are colored in black. The lower enclosed figure is a superposition of a drawing of five sections around the cell body of neuron #1 and arrowheads filled in magenta indicate the points of axonal endings of branches a-1 and c-1 in layer VI of PreS near the level of the cell body. Short double lines (magenta) indicate that the axon continues further, and a pound sign indicates an axonal branch entering the dorsal hippocampal commissure. Short double lines (magenta) on the axonal arbors of the whole drawing of neuron #1 indicate the same position as that of the enclosed figure. Scale bars = 5 mm in the upper right and 1 mm in the other photomicrographs of **(A)**, and 500 μm in the upper-middle and 100 μm in the upper-right and lower enclosed diagrams in **(B)**.

After a survival period of 72 h, rats were re-anesthetized by intraperitoneal injection of sodium pentobarbital (80 mg/kg body weight) and perfused transcardially with physiological saline followed by 4% formaldehyde in 0.1 M phosphate buffer (PB; pH 7.4), and the brains were removed. Brains were cut into several blocks and post-fixed with the same fixative for 4–5 h at 4°C. We created an “extended” hippocampal formation (Ishizuka, 2001; Honda and Ishizuka, 2004; Honda et al., 2008) to facilitate analysis of the laminar and topographical distributions of labeled axonal arbors and terminal boutons. In brief, cerebral hemispheres were dissected free from the diencephalon and gently flattened in fixative to reduce the natural concavity of the hippocampal formation and parahippocampal areas (Figure 1A). After cryoprotection with 20% sucrose in PB, transverse sections of the flattened hemisphere, perpendicular to the “extended” septotemporal (longitudinal) axis of the hippocampal formation, were cut at a thickness of 50 μm using a freezing microtome.

### Immunostaining for GFP

After confirming the presence of several GFP-expressing cells in sections containing PreS under a fluorescent microscope, all sections were immunostained with antibody against purified GFP. Sections were first incubated in PBS containing 0.1% hydrogen peroxide for 1 h at room temperature and washed in PBS. Sections were then incubated overnight at 4°C with anti-GFP antibody solution (1:500, A11120; Molecular Probes, Eugene, OR, USA; Table 2) containing 0.3% Triton X-100 and 1% normal donkey serum (NDS) in PBS. After rinses with PBS, sections were incubated in biotinylated anti-mouse IgG solution (1:100, AP192B; Millipore, Temecula, CA, USA; Table 2) containing 0.3% Triton X-100 and 1% NDS in PBS for 1 h at room temperature. Following a series of rinses with PBS, sections were incubated in avidin-biotin complex solution (Vectastain ABC Elite kit; Vector Laboratories, Burlingame, CA, USA). Sections were rinsed in PBS and placed in a solution of 0.05% 3,3′-diamininobenzidine-4HCl (DAB), 0.4% nickel (II) acetate, and 0.005% H_2_O_2_ in 50 mM Tris-HCl buffer (pH 7.6) for 30 min at room temperature. After several washes in Tris-buffered saline (TBS), all sections were serially mounted on gelatin-coated slides and counterstained with neutral red. Sections were then dehydrated in an ethanol series, cleared with xylene, and coverslipped.

**Table 2:** Antibodies used in this study.

Name	Host species	Clonality (Clone ID)	Source, Cat. #, RRID	Concentration used
Mouse anti-green fluorescent protein (GFP)	mouse	Monoclonal antibody (3E6)	Molecular Probes, A-11120, RRID:AB_221568	1:500
Donkey anti-mouse IgG, biotin-conjugated	donkey	Polyclonal antibody	Millipore, AP192B, RRID:AB_92624	1:100

### Reconstruction and Imaging of Labeled Neurons

Axons and dendrites of labeled neurons were first traced under an Eclipse 80i microscope (Nikon, Tokyo, Japan) attached with a camera lucida apparatus (at ×40 magnification). In the process of neuronal tracing, we labeled the axonal arbor by painting with several colors to identify each axonal branch originating from a single cell body. When there were any inexact points of joining and tracing fragments of axonal fibers, such as in cases where the target axonal branch was indistinguishable from other overlapping processes, we excluded these fibers from our dataset. Some neurons were also mapped and reconstructed using a computer-assisted microscope system and data analysis program (Neurolucida™; MicroBrightField, Colchester, VT, USA; at ×100 magnification). Shrinkage of section thicknesses was corrected along the z (depth) axis by Neurolucida™. In addition, two-dimensional unfolded maps were prepared as previously described (Honda and Ishizuka, 2004). Digital photomicrographs were taken using a LINCE™ (Claro, Aomori, Japan) with the extended focus application. The number of focus levels was 25, and the focus step size was 5 μm. Captured digital images were trimmed and adjusted to obtain optimal resolution, brightness, and contrast in Adobe Photoshop™ software (Adobe Systems, San Jose, CA, USA).

## Nomenclature

We have used the terms “septal-temporal” and “dorsal-ventral” interchangeably to represent the longitudinal direction of PreS (for details, see Ishizuka, 2001). We have also used the term “proximal-distal” to represent the transverse direction of PreS and EC instead of “anterior-posterior” and/or “medial-lateral.” In PreS, the term “proximal” means near Sub, whereas “distal” means distant from Sub. In EC, the term “proximal” means near ParS, whereas “distal” means near the rhinal fissure. The cutting plane and XY-direction of all traced images and photomicrographs in our figures were substantially the same. The so-called “postsubiculum” is treated as the septal part of PreS, on the basis of the strong similarities in connectivity and cytoarchitecture between the septal and temporal parts of PreS (Honda and Ishizuka, 2004; Honda et al., 2008). For purposes of description, we divided PreS into five portions along the septotemporal axis (septal, mid-septal, middle, mid-temporal, temporal), each about 1 mm long. According to this compartmentalization, PreS, septal PreS, mid-septal PreS, and the septal part of mid-PreS seem to correspond to the postsubiculum.

## Results

The cell bodies of infected neurons were diffusely distributed with sufficient distance between each other within a radius of about 1 mm from the center of injection. Consequently, we could reconstruct the whole shape of axonal processes originated from multiple virus-infected neurons within the same hemisphere of the same animal. In the present study, 54 presubicular neurons (including layers II–VI of PreS) were visualized in all animals of Table 1, of which 37 neurons (68.5%) were successfully reconstructed. In detail, 100% in layer II (4 neurons reconstructed/4 neurons visualized), 70.5% in layer III (12/17), 47.6% in layer V (10/21), and 100% in layer VI (8/8). The shortest distance between nearby virus-infected neurons was approximately 50 μm and the longest distance was 900 μm along the proximodistal or septotemporal axes of PreS. Twelve single layer-III presubicular neurons with labeled axons were successfully reconstructed in eight hemispheres ipsilateral to the injection site (Table 1). Six of the 12 neurons (including both pyramidal and non-pyramidal neurons) were cortical projection neurons that sent axons to MEC and/or Sub, or to ParS. The other six neurons were intrinsic projection-type neurons that sent many recurrent fibers to several layers of PreS but did not send efferents to other cortical areas.

### Cortical Projection Neurons

**Figure 2:**
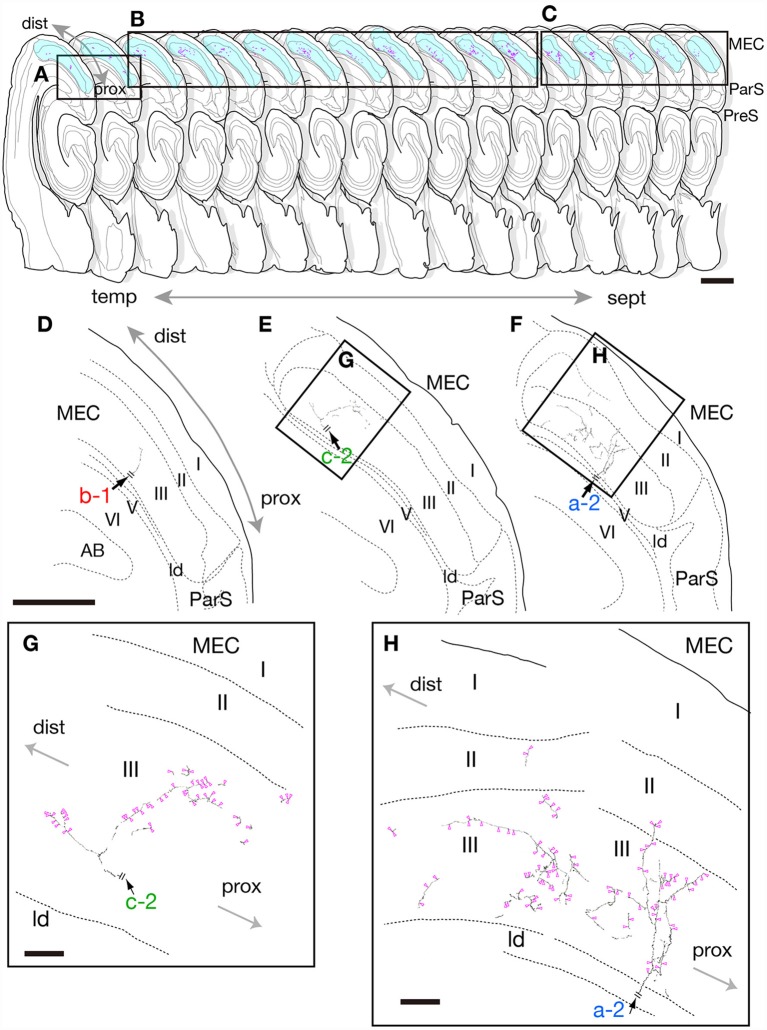
Drawing of 17 serial sections of the same hemisphere as neuron #1 are aligned in septotemporal order (upper) and camera lucida reconstruction of the terminal domain of each of axonal branches a-2, b-1 and c-2 in medial entorhinal cortex (MEC; middle and lower). Each axon is a continuation of the branches in Figure 1B. Short double lines (black) indicate the same position as in the whole drawing of neuron #1 in Figure 1B. Panels **(D–F)** are superpositions of drawings from two, 10 and five sections, i.e., **(A–C)**, respectively, as area arranged from temporal **(D)** to septal **(F)** levels. Higher-powered magnification of the rectangles in **(E,F)** are indicated as **(G,H)**, respectively. Panels **(G,H)** are superpositions of the drawing of five or seven sections using a ×100 objective lens. Magenta-open arrowheads indicate the position of identifiable axonal boutons. Scale bars = 1 mm in the upper row, 500 μm in the middle row **(D–F)** and 50 μm in **(G,H)**.

**Figure 3:**
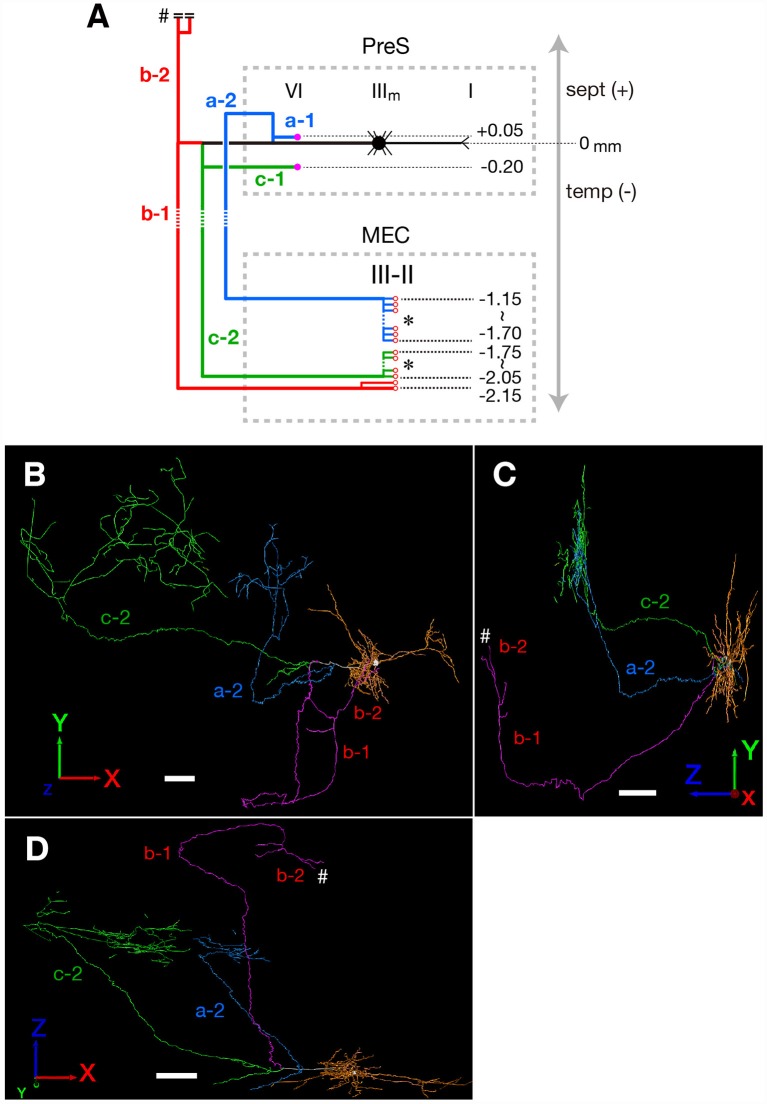
**(A)** Schematic diagram of the axonal branching pattern for neuron #1. The main axon that comes directly out of the cell body is colored in black, and the other axonal branches are represented using the same colors as in Figure 1B. The dendrites and cell body are represented schematically as thin black lines and a black filled circle. Small red-open circles indicate terminal levels of axonal branches within layers II–III of MEC and asterisks near those terminals indicate the existence of complex terminal arborizations that form the plexus. Numbers indicate the septotemporal levels of terminations for each axon collateral when the level of cell body is set as the zero-point. Short double lines and a pound sign at the upper end of branch b-2 indicate that the nerve continues farther through the dorsal hippocampal commissure. Dotted rectangles indicate areas of PreS and MEC. IIIm in PreS represents the middle depth of layer III of PreS. The level of each axonal branching point is not necessarily accurate. **(B–D)** Images of 3D-reconstructed neuron #1 are indicated; a frontal view from the z-axial direction **(B)**, a view of the right side from the x-axial direction **(C)** and a view of the bottom from the y axial direction **(D)**. The z-axis corresponds to septal and temporal (longitudinal axis of the hippocampal formation). In PreS, the x-axis corresponds to superficial and deep, and the y-axis to proximal (near Sub) and distal. In EC, the x axis corresponds to proximal (near ParS) and distal (near rhinal fissure), and the y-axis to superficial and deep. The cell body is represented as a white circle and the dendrites are colored as orange. The main axon that comes directly out of the cell body is colored in white, and the other axonal branches are represented using the same colors as in Figure 1B. White pound signs at the end of branch b-2 in **(C,D)** indicate that the branch enters into the dorsal hippocampal commissure and continues farther. In comparison with Figure 1B, higher magnification is used in the 3D-reconstruction and the number and volume of terminal arbors appear to be increasing in (**B–D**; see “Materials and Methods” section). Scale bars = 100 μm in **(B–D)**.

**Figure 4:**
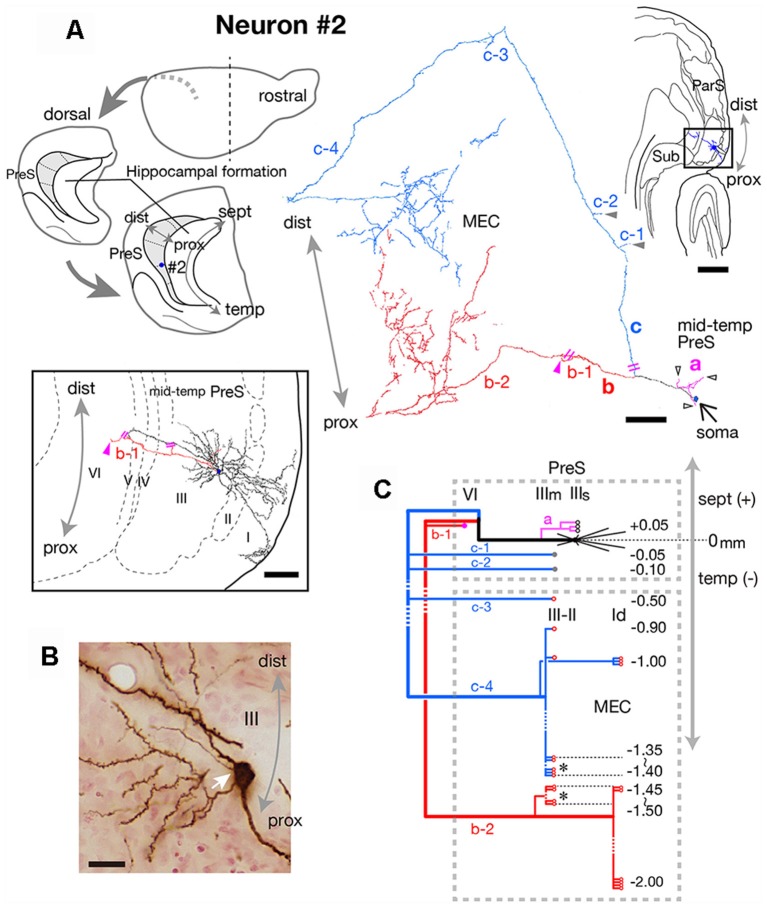
**(A)** Schematic diagrams of the extended hippocampus as shown in Figure 1A (upper left). The localization of cell body of neuron #2 is represented as a blue-colored circle. A drawing of the section that includes the cell body and several dendrites (upper right) and camera lucida reconstruction of axonal morphology (upper middle) of neuron #2. The cell body is painted over in blue. The three main axonal branches a, b and c and their collaterals are represented by magenta, red and blue, respectively. The main axons that come directly out of the cell body are colored in black. The black-open, gray and magenta arrowheads indicate points of axonal endings in the superficial part of layer III, middle part of layer III, and layer VI of PreS, respectively. Indications of axonal ending points in MEC are omitted from this drawing. The lower enclosed figure is a superposition of a drawing of sections around the cell body of neuron #2 and arrowheads filled in magenta indicate the points of axonal endings of branch b-1 in layer VI of PreS near the level of the cell body. Short double lines (magenta) indicate that the axon continues further. Short double lines (magenta) on the axonal arbors of the whole drawing of neuron #2 indicate the same position as that of the enclosed figure. **(B)** Composite photomicrograph of the cell body and proximal parts of its processes taken by stacking different focal planes. White arrow indicates the point of origin of the main axon, which comes directly out of the cell body. **(C)** Schematic diagram of the axonal branching pattern of neuron #2. The axonal branches are represented using the same colors as in **(A)**. Black-open, gray and magenta circles indicate terminal levels of axonal branches within the superficial part of layer III, middle part of layer III and layer VI of PreS, respectively. Red-open circles indicate terminal levels of axonal branches within the superficial layers of MEC. Asterisks near those terminals indicate the existence of complex terminal arborizations that form a plexus. Numbers indicate septotemporal levels of terminations for each axon collateral when the level of the cell body is set as the zero-point. IIIs and IIIm in PreS represent the superficial and middle depths of layer III of PreS. The level of each axonal branching point is not necessarily accurate. Scale bars = 500 μm in the upper-right and 100 μm in the middle and lower enclosed diagrams in **(A)**, and 20 μm in **(B)**.

**Figure 5:**
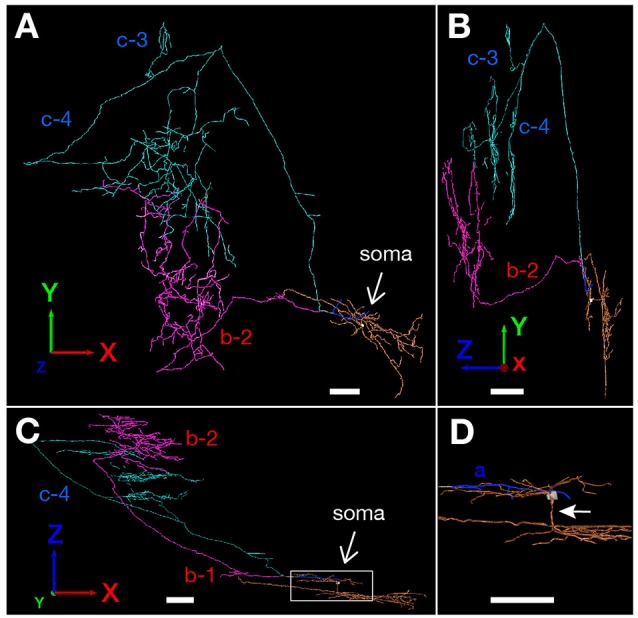
The 3D-reconstructed images of neuron #2 are indicated: a frontal view from the z-axial direction **(A)**; a view of the right side from the x-axial direction **(B)**; and a view of the bottom from the y-axial direction **(C)**. The cell body and main axon that comes directly out of the cell body are colored in white, and the three main axonal branches a, b and c and their collaterals are represented by blue, magenta and pale blue, respectively. Dendrites are colored as orange. **(D)** High magnification of the rectangle in **(C)**. White arrow indicates a shaft of apical dendrite. Scale bars = 100 μm in **(A–D)**.

**Figure 6:**
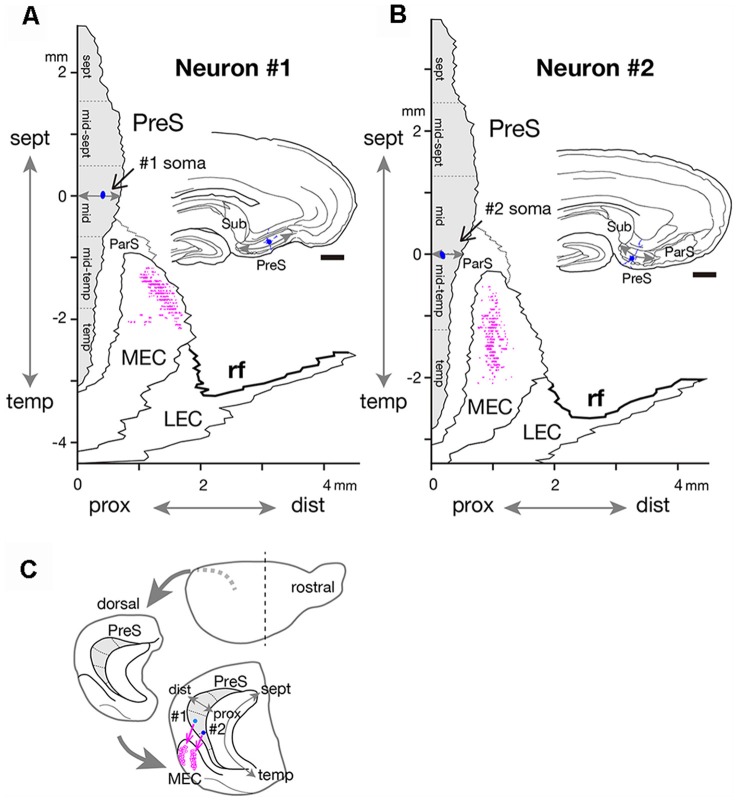
Two-dimensional unfolded maps of layer III of PreS and MEC with surrounding areas, showing the locations of the cell body (blue filled circles) and axon terminals (magenta dots) of neurons #1 **(A)** and #2 **(B)**. The vertical axis of the map indicates the distance from the septotemporal level of the cell body, and the horizontal axis indicates the distance from the proximal end of PreS. For details, see Honda and Ishizuka (2004) and Honda et al. (2008). Drawings of the sections for neurons #1 and #2, which include the cell body (blue circle) and several dendrites (blue short lines) are inserted in each unfolded map. A gray double arrow in each drawing indicates the proximodistal length of PreS. **(C)** Schematic diagrams of the extended hippocampus as shown in Figure 1A. The location of cell body in PreS is represented as light blue- (neuron #1) and blue- (neuron #2) colored circles and the terminal distribution in layers II–III of MEC are schematically shown by magenta-open circles. Scale bars = 500 μm in **(A,B)**.

**Figure 7:**
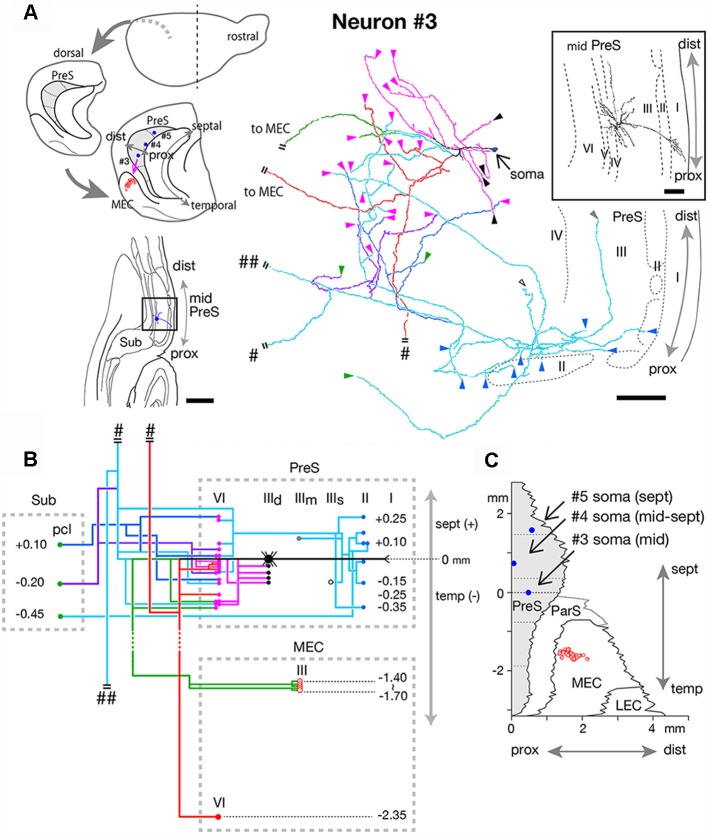
**(A)** Schematic diagrams of the extended hippocampus as shown in Figure 1A (upper left). The locations of cell body of neurons #3, #4, and #5 in PreS are schematically represented as blue-colored circles. The terminal distribution in layers II–III of MEC originated from neuron #3 is schematically shown by red-open circles. Lower left is a drawing of the section of neuron #3, which includes the cell body and several dendrites. Camera lucida reconstruction of axonal morphology of neuron #3 (middle). The enclosed figure is a superposition of drawings of the soma and dendrites. The cell body is painted over in blue. The main axonal branches and their collaterals are represented by magenta, red, green and pale blue. One of the collaterals from the pale blue branch is colored in blue and the other in purple. Short double lines indicate that the axon continues farther. The points of axonal endings in layer II of PreS are indicated as blue arrowheads. Axonal endings in the superficial, middle and deep depths of layer III of PreS are indicated as open, gray and black arrowheads, respectively. Magenta arrowheads indicate points of endings in layer VI of PreS. **(B)** Schematic diagram of the axonal branching pattern of neuron #3. The axonal branches are represented using the same colors as in **(A)**. Blue, black-open, gray, black and magenta circles indicate terminal levels of axonal branches within the superficial, middle, and deep parts of layer III and layer VI of PreS, respectively. Red-open and red-colored circles indicate terminal levels of axonal branches within layers III and VI of MEC, respectively. IIId in PreS represents the deep part of layer III of PreS. The level of each axonal branching point is not necessarily accurate. Short double lines indicate that the axon continues farther. Single and double pound signs in **(A,B)** indicate that the axonal branches run into the corpus callosum and fimbria/fornix, respectively. **(C)** Two-dimensional unfolded map of layer III of PreS (shaded region), ParS and EC, showing the locations of the cell body (blue filled circles) of neurons #3, #4, and #5 and axon terminals (red-open circles) of the green branch of neuron #3 in layer III of MEC. Scale bars = 500 μm in the lower-left and 100 μm in the right and enclosed diagrams in **(A)**.

**Figure 8:**
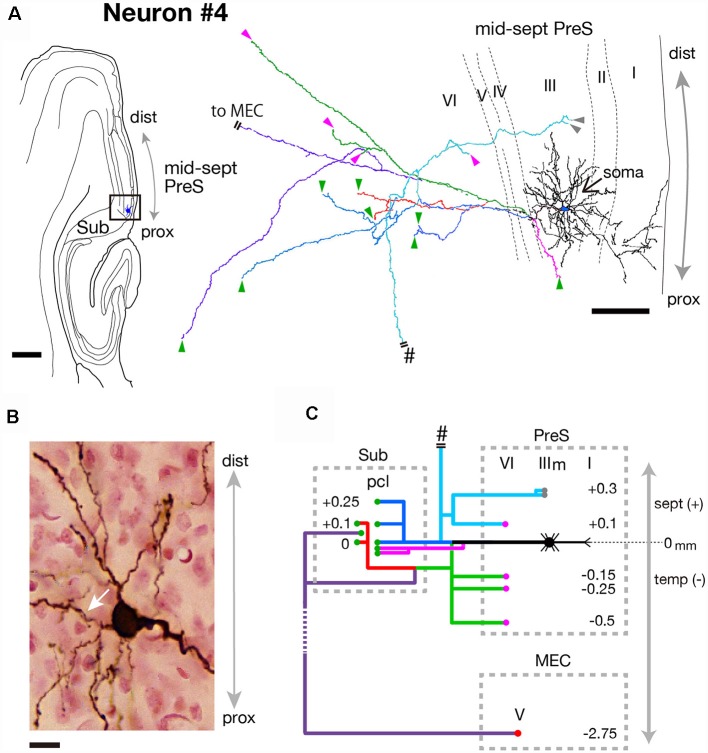
**(A)** A drawing of the section which includes the cell body and several dendrites (left) and camera lucida reconstruction of the axonal morphology of neuron #4 (right). The cell body is painted over in blue. The main axonal branches and their collaterals are represented by blue, magenta and green. One of the collaterals from the blue branch is colored in pale blue and two branches from green are colored in red and purple. Short double lines indicate that the axon continues farther. The points of axonal endings at the middle depth of layer III of PreS and in layer VI of PreS are indicated as gray and magenta arrowheads, respectively. Axonal endings in the pyramidal cell layer of Sub are indicated as green arrowheads. **(B)** Composite photomicrograph of the cell body and proximal parts of its processes taken by stacking different focal planes. A white arrow indicates the main axon. **(C)** Schematic diagram of the axonal branching pattern for neuron #4. The main axon is colored in black, and the other axonal branches are represented using the same colors as in **(A)**. Gray- and magenta-colored circles indicate terminal levels of axonal branches within the middle part of layer III and layer VI of PreS, respectively. Green- and red-colored circles indicate terminal levels of axonal branches within the pyramidal cell layer of Sub and layer V of MEC, respectively. The level of each axonal branching point is not necessarily accurate. Short double lines indicate that the axon continues farther. Pound signs in **(A,C)** indicate that the axonal branches run into the corpus callosum. Scale bars = 500 μm in the left and 100 μm in the right in **(A)** and 10 μm in **(B)**.

**Figure 9:**
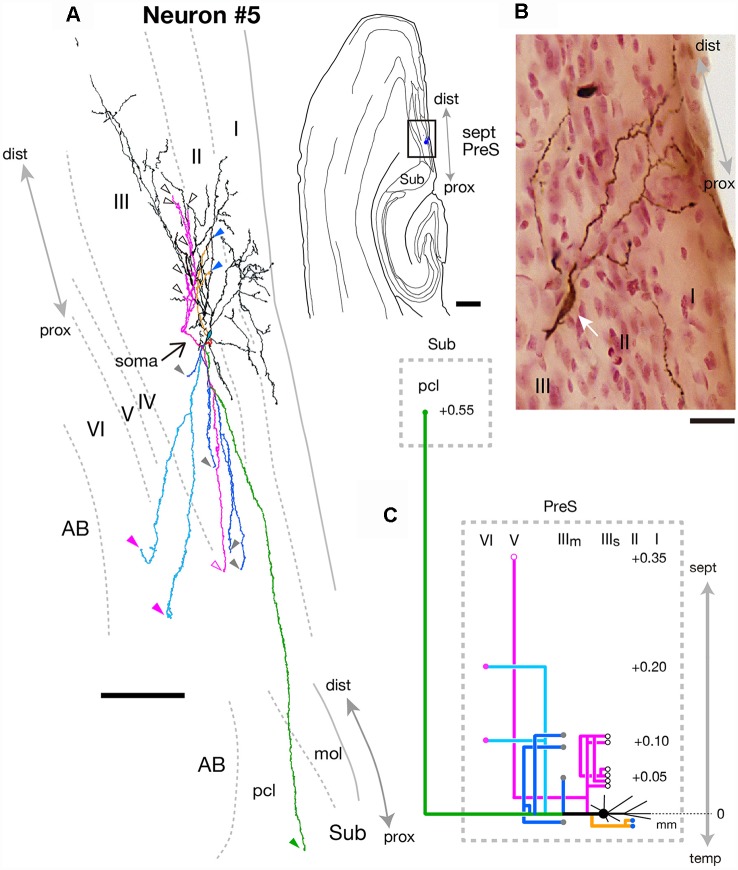
**(A)** A drawing of the section which includes the cell body and several dendrites (right) and camera lucida reconstruction of axonal morphology of neuron #5 (left). Dendrites and the main axon are colored in black. The main axonal branches and their collaterals are represented by orange, magenta and green. One of the collaterals from the green branch is colored in pale blue and three branches from the green branch are colored in blue. Points of axonal endings in layer II, the superficial and middle depths of layer III, layer V and layer VI of PreS are indicated as blue, white-open, gray, magenta-open and magenta arrowheads, respectively. The point of axonal ending in the pyramidal cell layer of Sub is indicated as a green arrowhead. **(B)** Composite photomicrograph of the cell body and proximal parts of its processes taken by stacking different focal planes. White arrow indicates the spindle-shaped cell body. **(C)** Schematic diagram of the axonal branching pattern for neuron #5. The axonal branches are represented using the same colors as in **(A)**. Blue, white-open, gray, magenta-open and magenta circles indicate terminal levels of axonal branches within layer II, the superficial and middle parts of layer III, layer V and layer VI of PreS, respectively. A green circle indicates the terminal level of axonal branches within the pyramidal cell layer of Sub. The level of each axonal branching point is not necessarily accurate. Scale bars = 500 μm in the right and 100 μm in the left in **(A)** and 20 μm in **(B)**.

**Figure 10:**
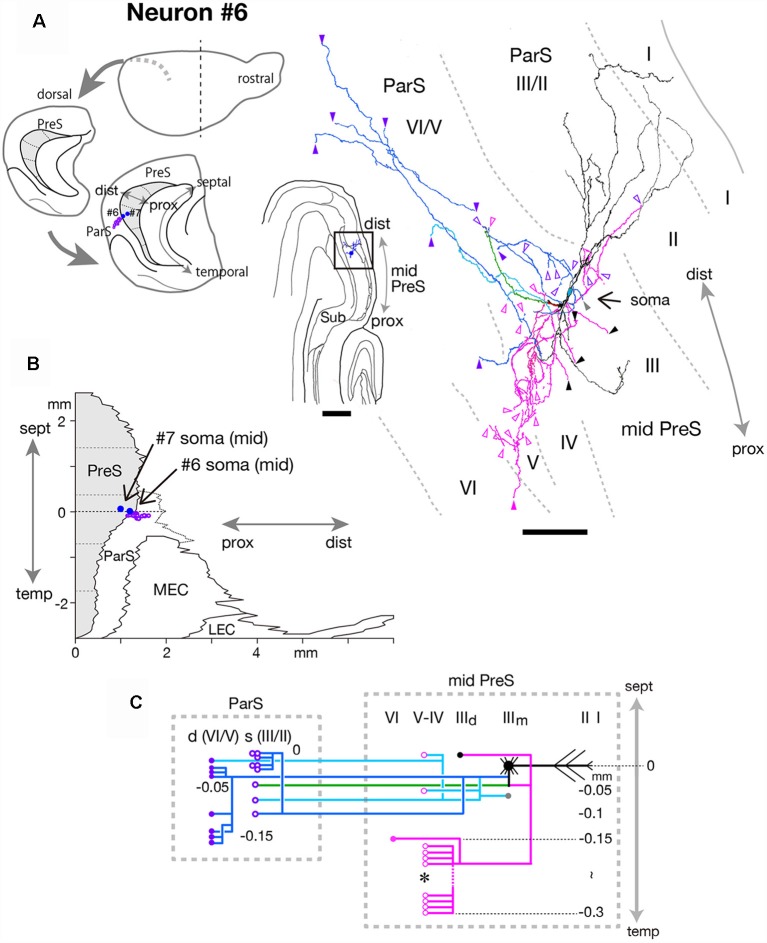
**(A)** Schematic diagrams of the extended hippocampus as shown in Figure 1A (left). The locations of cell body of neurons #6 and #7 in PreS are schematically represented as blue-colored circles. The terminal distribution in the superficial layers of ParS originated from neuron #6 is schematically shown by purple-open circles. Middle is a drawing of the section of neuron #6, which includes the cell body and several dendrites. Camera lucida reconstruction of axonal morphology of neuron #6 (right). Dendrites and the main axon are colored in black, and the main axonal branches and their collaterals are represented by blue, magenta and green. One of the collaterals from the blue branch is colored in pale blue. Gray, black, magenta-open and magenta arrowheads indicate the points of ending of axonal branches in the middle and deep parts of layer III, layer V and layer VI of PreS, respectively. Purple-open and purple arrowheads indicate points of endings of axon collaterals in the superficial (layers II/III) and deep (layers V/VI) layers of ParS, respectively. **(B)** Two-dimensional unfolded map of layer III of PreS, ParS and EC, showing the locations of the cell body (blue-colored circles) of neurons #6 and #7 and axon terminals (purple-open circles) of neuron #6 in the superficial layers of ParS. **(C)** Schematic of axonal branching pattern for neuron #6. The axonal branches are represented using the same colors as in **(A)**. Gray, black, magenta-open and magenta circles indicate terminal levels of axonal branches within the middle and deep parts of layer III, layer V and layer VI of PreS, respectively. Purple-open and purple circles indicate terminal levels of axonal branches within the superficial (layers II/III) and deep (layers V/VI) layers of ParS, respectively. An asterisk indicates the presence of complex terminal arborization forming a plexus. The level of each axonal branching point is not necessarily accurate. Scale bar = 500 μm in the middle and 100 μm in the right in **(A)**.

Within the six cortical projection neurons, two sent fibers only to MEC (neurons #1 and #2; Figures 1–6) and another two sent axon collaterals to both MEC and Sub (neurons #3 and #4; Figures 7, 8). One of the remaining two neurons projected only to Sub (neuron #5; Figure 9), and the other provided numerous branches only to ParS (neuron #6; Figure 10). All neurons had two or more recurrent collaterals, which terminated in one or more layers of PreS (Table 1).

#### MEC Projection Neurons

Neuron #1 was a pyramidal neuron with the cell body located at the middle depth of layer III of the mid-proximodistal part of mid-PreS (Figures 1B, 6A). This neuron gave off three major axonal branches (a, b and c in Figure 1B), with their three collaterals (a-2, b-1 and c-2 in Figures 1B, 2, 3) reaching layers II and III of MEC. As seen in Figure 2, different patterns of termination were seen in MEC, i.e., branches a-2 and c-2 provided complex terminal arborizations with many en passant boutons, while branch b-1 showed no terminal arborizations in layer III of MEC. While ascending in layer III of MEC, branch b-1 formed several en passant boutons (not shown). Both branches a-2 and c-2 formed elaborate terminal arbors that seemed to face each other, while their terminal distributions rarely overlapped within layer III of MEC (Figures 3B,D). To clarify the positional relationship between the soma and axon terminals, we created representations of these structures on an unfolded map of the entire PreS and EC. The area of termination of neuron #1 showed a band-like pattern, with the transverse axis disposed parallel to the rhinal fissure and the longitudinal axis perpendicular to the boundary between MEC and LEC (Figure 6A). This band-like field was about 0.5 mm in width and located in the distalmost region of MEC. A small cluster of terminals was seen at the more proximal part of MEC, corresponding to terminals derived from branch b-1. From branch b, an additional axonal branch entered the dorsal hippocampal commissure (b-2 in Figures 1B, 3). Both branches a and c showed a short recurrent collateral, which reached layer VI of PreS near the cell body (a-1 and c-1 in Figures 1B, 3A) and they formed small terminal boutons on branch endings (not shown).

Neuron #2 was an atypical pyramidal neuron (Figure 4A), with an apical dendrite (arrow in Figure 5D) directed more temporally to the cell body and giving off branches approximately parallel to the plane perpendicular to the septotemporal axis of PreS. The soma was located at the superficial part of layer III in the mid-proximodistal portion of the mid-temporal PreS (Figures 4A, 6B). An axon emanating directly from the cell body (white arrow in Figure 4B) sent a short recurrent collateral to the superficial part of PreS near the cell body (branch a in Figures 4A,C), then bifurcated into two major axonal branches b and c toward the superficial layers of MEC (Figures 4A,C). After providing a recurrent collateral b-1 to layer VI of PreS, which formed several en passant boutons within layer VI (not shown), branch b-2 ran into layer VI of MEC, then vertically ascended to the superficial layers to form a complex terminal arborization within layer III (Figures 4A,C, 5). Branch c had two recurrent collaterals, c-1 and c-2, both terminating in the middle part of layer III of PreS. One collateral (c-3) ran through the lamina dissecans of MEC and terminated in layer III of MEC without any complex terminal arborizations (Figures 4A,C, 5). On the other hand, branch c-4 arborized extensively within the superficial layers through almost 0.5 mm along the septotemporal axis of MEC (Figure 4C) and formed a complex terminal arborization at the level adjoining the b-2 axon terminal ramification (asterisks in Figure 4C). Similar to neuron #1, plexuses of the b-2 and c-4 terminal arborizations expanded along the proximodistal axis and seemed to face each other within the superficial layers of MEC, while the terminal distributions rarely overlapped (Figures 4A, 5). Both branches b-2 and c-4 sent one axon collateral to the deep part of layer I of MEC that reached levels separate from the terminal arborizations in layers II–III (Figure 4C). As seen in the unfolded map (Figure 6B), axon terminals in layer III of MEC distributed in a band-like fashion similar to the terminal distribution pattern of neuron #1 (Figure 6A). Compared with neuron #1, the band-like terminal region of neuron #2 was located more proximally in MEC (Figures 6A,B), probably because the cell body of neuron #2 was situated at a more temporal level of PreS than the cell body of neuron #1 (Figure 6C). No commissural axonal branches ascending in the white matter could be found for neuron #2.

In conclusion, neuron #1 was a pyramidal neuron, and neuron #2 was an atypical pyramidal neuron, both had two axonal branches which formed a complex terminal arborization within the superficial layers of MEC.

#### MEC/Sub Projection Neurons

Neuron #3 was a spiny pyramidal neuron, and the locus of the cell body was in the deep part of layer III in the mid-proximodistal portion of mid-PreS (Figure 7A). This neuron provided four types of axonal branches, classified by the terminal regions. The first group comprised branches that had several short recurrent collaterals terminating in layers III or VI of PreS (magenta branches in Figures 7A,B), and the second consisted of axonal branches reaching layer III of MEC and also supplying several recurrent collaterals to layer VI of PreS (green branches in Figures 7A,B). The third group represented branches bifurcating into collaterals projecting to layer VI of MEC and to the contralateral hemisphere through the corpus callosum (red branches in Figures 7A,B), and also showing two short recurrent collaterals terminating in layer VI of PreS. The last group comprised branches reaching the pyramidal cell layer of Sub (blue, pale blue and purple branches in Figures 7A,B), which provided many recurrent collaterals to layers II, III and VI of PreS. The complex terminal arborizations within the superficial layers of the proximal (near Sub) part of PreS derived from a single axonal branch that also provided collaterals to layer VI of PreS and to Sub (pale blue branches in Figures 7A,B). Interestingly, terminations of the three axonal branches within Sub distributed at similar intervals of 0.25–0.3 mm along the septotemporal axis (Figure 7B). Moreover, one such branch bifurcated into collaterals to the contralateral hemisphere through the corpus callosum and to subcortical areas *via* the fimbria-fornix (pound sign and double pound sign on pale blue branches in Figures 7A,B). All axonal arbors which terminated in layer VI of PreS and MEC formed small terminal boutons in their target layer. As seen in the unfolded map (Figure 7C), the proximodistal range of the terminal distribution was about 500 μm, similar to the band-like terminal regions of neurons #1 and #2 (as shown in Figure 6), but the septotemporal length was significantly shorter than those of neurons #1 and #2.

Neuron #4 was an atypical pyramidal cell with a curved apical dendrite that ran obliquely across layer I and reached the pial surface (Figure 8A). Dendritic spines were relatively sparse (Figure 8B). The cell body was located at the middle depth of layer III of the proximal portion of the mid-septal PreS (Figures 7A,C), and provided many axonal arbors to Sub (Figure 8C). Four major branches (magenta, green, blue and pale blue branches in Figures 8A,C) were sent out from the main axonal shaft (white arrow in Figure 8B), each showing collaterals that terminated in the pyramidal cell layer of Sub. One of the major branches (pale blue branch in Figure 8C) ascended to the septal levels and bifurcated into recurrent collaterals to layers III and VI of PreS and a commissural axonal branch, which entered the dorsal hippocampal commissure (pound signs in Figures 8A,C). A descending axonal arbor (green branch in Figures 8A,C) was also present, sending collaterals to both layer V of MEC and the pyramidal cell layer of Sub (red and purple branches in Figures 8A,C), in addition to three recurrent collaterals to layer VI of PreS. All axonal arbors which terminated in the deep layers of PreS and MEC formed small terminal boutons in their target layers.

Taken together, two distinct types of MEC/Sub projection neurons were observed; neuron #3 was a spiny pyramidal neuron, which had both commissural and subcortical projection fibers and also provided many recurrent collaterals to various layers of PreS, while neuron #4 was an atypical pyramidal neuron, which had relatively sparse recurrent collaterals and provided more axonal arbors to Sub than to MEC.

#### Subicular Projection Neuron

As seen in Figure 9, neuron #5 was a subicular projection neuron, which had only one axonal arbor to Sub. The spindle-shaped cell body (white arrow in Figure 9B) was located in the superficial part of layer III of the mid-proximodistal portion of the septal PreS (Figures 7A,C). The form of dendritic arborization extended widely to the more temporal levels for about 0.5 mm along the septotemporal axis (not shown) within layers I–III of PreS (Figure 9A). Many of the axonal collaterals projected to levels more septal than the cell body. One of the main branches showed a collateral that terminated in layer V of PreS at a level 0.35 mm more septal than the cell body, while many shorter recurrent collaterals terminated within the superficial part of layer III (magenta branches in Figures 9A,C). Branches were seen specifically projecting to layer II (yellow branch in Figures 9A,C) or layer VI (pale blue branch in Figures 9A,C) of PreS, and the latter bifurcated into an axonal arbor reaching to Sub at a level 0.55 mm more septal than the cell body (green branch in Figures 9A,C). All remaining branches innervated the middle depth of layer III of PreS (blue branches in Figures 9A,C).

In summary, neuron #5 was a fusiform cell, which provided one axon collateral to Sub in addition to many recurrent collaterals to PreS itself.

#### Parasubicular Projection Neuron

At the level of mid-PreS, an elongated region was seen that seemed to be the septal-most part of ParS. Our previous study also confirmed that this region was not included in the intrinsic connectivity of PreS (Honda et al., 2008). The fusiform-shaped cell body of neuron #6 was located at the middle depth of layer III of the distal portion of mid-PreS and provided many axonal collaterals specifically to the septal-most part of ParS (Figure 10A). Ascending dendritic arbors were spiny and reached to layers I and II/III of ParS, and the descending dendrites reached to layer III of the distal part of PreS. This neuron seemed to be a bitufted type, as defined by the form of dendritic arborization, while the axonal arbors extended horizontally from the distal edge of PreS to several layers of the proximal part of ParS. We plotted precise localizations of soma and the axon terminals (purple-open arrowheads in Figure 10A and purple-open circles in Figure 10C) of neuron #6 on the unfolded map as shown in Figure 10B. The termination of the blue, pale blue and green axons of neuron #6 (Figures 10A,C) was localized in the proximal region of the septal-most part of ParS (Figure 10B). The main axonal shaft branched into two major collaterals (blue and green branches in Figures 10A,C), which mostly innervated ParS and also provided one major collateral for intrinsic projection (magenta branch in Figures 10A,C). From one of the two major collaterals to ParS (blue branch in Figures 10A,C), two branches bifurcated, each specifically terminating in the superficial layers (layers II/III) or deep layers (mostly layers IV–V, including lamina dissecans) of ParS. This major branch also displayed several short recurrents to layers III and V near the level of the cell body (pale blue branches in Figures 10A,C). A major axonal branch for intrinsic connection (magenta branch in Figures 10A,C) bifurcated into two collaterals, one of which terminated in the deep part of layer III while the other descended for about 0.2 mm temporal from the cell body, then formed an intricate terminal arborization within layers IV–V of PreS (asterisk in Figure 10C).

Taken together, these findings indicate neuron #6 was a fusiform cell, which sent many axonal arbors to ParS and also had recurrent axon collaterals for dense intrinsic connections within PreS.

### Intrinsic Connectional Neurons

**Figure 11:**
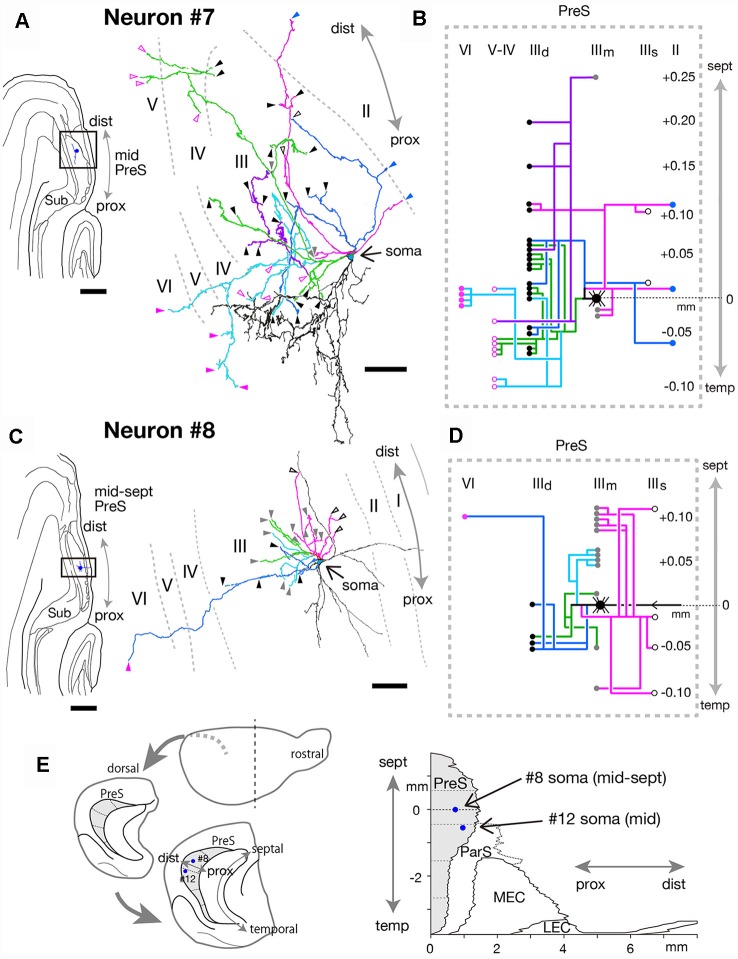
**(A,C)** A drawing of the section which includes the cell body and several dendrites (left) and camera lucida reconstruction of axonal morphology (right) of neurons #7 **(A)** and #8 **(C)**. Dendrites are colored in black. The main axonal branches and their collaterals are represented by magenta, blue, green (in **A**) and pale blue (in **C**). In **(A)**, one of the collaterals from the green branch is colored in purple and the other in pale blue. Blue arrowheads indicate points of endings of axonal branches in layer II of PreS. White-open, gray, black, magenta-open and magenta arrowheads indicate the points of endings of axonal branches in the superficial, middle and deep parts of layer III, layer V and layer VI of PreS, respectively. **(B,D)** Schematic diagrams of each axonal branching pattern for neurons #7 **(B)** and #8 **(D)**. The axonal branches are represented using the same colors as in **(A,C)**. Blue, white-open, gray, black, magenta-open and magenta circles indicate terminal levels of axonal branches within layer II, the superficial, middle and deep parts of layer III, layer V and layer VI of PreS, respectively. **(E)** Schematic diagrams of the extended hippocampus as shown in Figure 1A (left). The locations of cell body of neurons #8 and #12 in PreS are represented as blue-colored circles. Two-dimensional unfolded map of layer III of PreS, ParS and EC, showing the locations of the cell body (blue-colored circles) of neurons #8 and #12 are inserted in (**E**, right). Scale bars = 500 μm in the left and 50 μm in the right in **(A,C)**.

**Figure 12:**
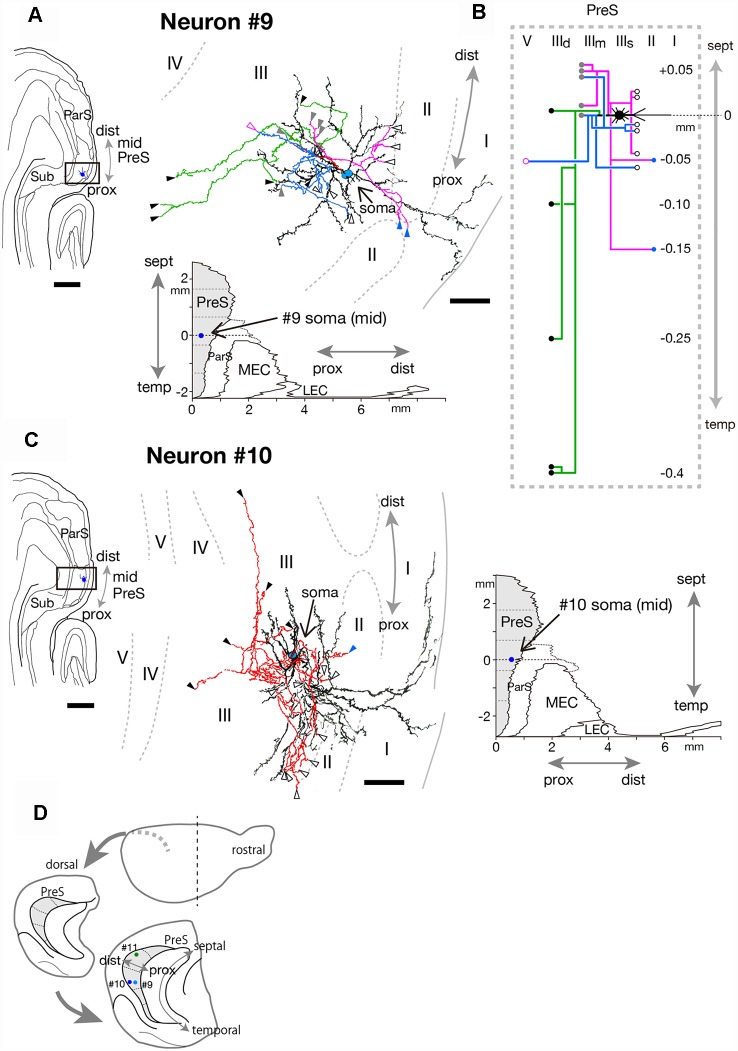
**(A,C)** A drawing of the section which includes the cell body and several dendrites (left) and camera lucida reconstruction of axonal morphology (right) of neurons #9 **(A)** and #10 **(C)**. Dendrites are colored in black. The main axonal branches and their collaterals are represented by magenta, blue and green in **(A)**. In **(C)**, all axons are colored in red. Blue, white-open, gray, black, magenta-open and magenta arrowheads indicate the points of endings of axonal branches in layer II, the superficial, middle and deep parts of layer III, layer V and layer VI of PreS, respectively. In **(C)**, some axon terminals are not denoted because of overlapping arbors. **(B)** Schematic diagrams of each axonal branching pattern for neuron #9. The axonal branches and the terminal levels are represented using the same colors as in **(A)**. The level of each axonal branching point is not necessarily accurate. **(D)** Schematic diagrams of the extended hippocampus as shown in Figure 1A. The locations of cell body of neurons #9, #10, and #11 in PreS are schematically represented as pale blue-, blue-, and green-colored circles, respectively. Scale bars = 500 μm in the left and 50 μm in the right in **(A,C)**.

Within six intrinsic projection neurons, four gave off many axonal branches reaching to both superficial and deep layers of PreS (Figure 11), while the other two displayed sparse axonal branches, for which terminations were confined to layers III–V or restricted within layer III of PreS (Figure 12).

#### Neurons With Numerous Axonal Collaterals

The cell body of neuron #7 was located at the middle depth of layer III of the distal portion of mid-PreS, which was at a distance of 50 μm from the cell body of neuron #6 (in the same animal; Figures 10A,B, 11A,B). The dendrites were spiny and showed a unique arborization pattern, arborizing extensively in a direction deep to the cell body (Figure 11A). A main axonal shaft provided three major branches (magenta, green and blue branches in Figures 11A,B), one of which formed complex terminal arborizations within the deep part of layer III near the level of the cell body (green branches in Figures 11A,B), with a collateral sending many branches to layers V and VI (pale blue branches in Figures 11A,B) and another collateral providing several axonal branches to the middle and deep parts of layer III from 0.05 mm temporal to 0.25 mm septal to the cell body (purple branch in Figures 11A,B). The other two major branches (magenta and blue branches in Figures 11A,B) gave off ascending collaterals to layer II and the superficial part of layer III, and also bifurcated several branches to the deep part of layer III.

Neuron #8 also showed characteristic dendritic arborization, i.e., one dendritic arbor spread in distal and superficial directions and the other mostly extending in a proximal direction from the cell body (Figure 11C). Dendrites of this neuron were relatively thin and smooth. The soma was located at the middle depth of layer III of the mid-proximodistal part of the mid-septal PreS (Figures 11C,E) and gave off four major axonal branches (magenta, blue, pale blue and green branches in Figures 11C,D), with all terminating within about 0.2 mm of the septotemporal range. One major branch sent many collaterals to a level 0.1 mm septal to the cell body and fewer to more temporal levels, and all collaterals terminated in the superficial or middle depth of layer III (magenta branches in Figures 11C,D). One of the other major branches specifically projected to the middle depth of layer III at a more septal level (pale blue branches in Figures 11C,D), while the other two provided collaterals mostly to the middle and deep parts of layer III at the level of the cell body or more temporal levels (blue and green branches in Figures 11C,D). One descending axonal arbor bifurcated from the major branch (blue branches in Figures 11C,D) and terminated at the deep part of layer VI.

Cell bodies of neurons #9 and #10 were located at the superficial part of layer III in the proximal and distal portions of mid-PreS, respectively (Figures 12A,C,D; in different animal). The axonal branches formed a local plexus at levels near the cell body and terminated in layers II, III and V. In addition, neuron #9 had long descending axonal branches (green branches in Figures 12A,B) that reached to a level about 0.4 mm temporal to the cell body.

In conclusion, neurons #7, #8, #9 and #10 had many recurrent axon collaterals, which mainly terminated in the deep and/or superficial part of layer III of PreS. Neurons #7 and #8 had asymmetric axonal domains, which was offset in the more distal part and deeper layers in neuron #7, while in the more distal and superficial part of layer III in neuron #8.

#### Neurons With Sparse Axonal Branches

**Figure 13:**
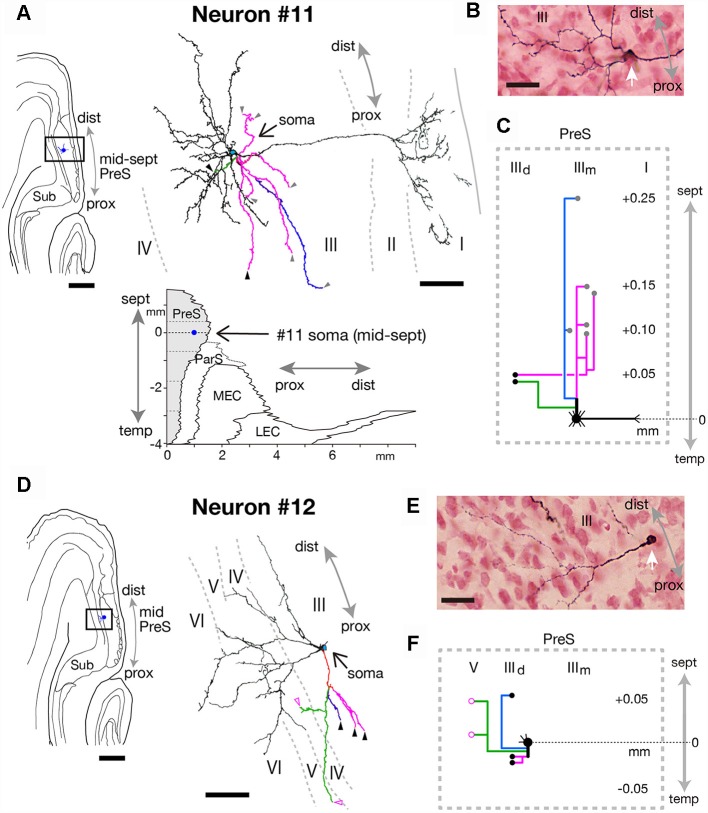
**(A,D)** A drawing of the section which includes the cell body and several dendrites (left) and camera lucida reconstruction of axonal morphology (right) of neurons #11 **(A)** and #12 **(D)**. A two-dimensional unfolded map of layer III of PreS (shaded region), ParS and EC, showing the locations of the cell body (blue filled circles) of neuron #11 is inserted in **(A)**. In the camera lucida reconstruction, dendrites are colored in black. The main axonal branches and their collaterals are represented by magenta, blue, and green. Gray, black and magenta-open arrowheads indicate points of endings of axonal branches in the middle and deep parts of layer III and layer V of PreS, respectively. **(B,E)** Composite photomicrographs of each cell body and proximal parts of the processes of neurons #11 **(B)** and #12 **(E)** taken by stacking different focal planes. White arrows indicate cell bodies. **(C,F)** Schematic diagrams of each axonal branching pattern of neurons #11 **(C)** and #12 **(F)**. The axonal branches and the terminal levels are represented using the same colors as in **(A,D)**. The level of each axonal branching point is not necessarily accurate. Scale bars = 500 μm in the left and 50 μm in the right in **(A,D)**, and 20 μm in **(B,E)**.

The cell bodies of neurons #11 and #12 were both located at the middle depth of layer III of the distal part of PreS, but with slightly different septotemporal distributions. The cell body of neuron #12 was located in mid-PreS, while that of neuron #11 was located in mid-septal PreS (Figures 11E, 12D, 13A). Both neurons provided a small number of axonal branches, for which the terminal distributions were confined to layer III (in neuron #11) or layers III and V (in neuron #12; Figure 13). Neuron #11 was a pyramidal neuron with spiny dendritic arbors (Figures 13A,B) and axonal branches mostly innervating the middle depth of layer III only at levels more septal to the cell body (Figure 13C). On the other hand, neuron #12 sent axons to more proximal and deeper parts than the cell body (Figures 13D,F). The dendrites were thin and displayed many small spines, particularly at the part distant from the cell body (Figure 13E). The dendritic morphology of neuron #12 showed a unique pattern, with dendritic arbors proximodistally expanding to the deeper layers (Figure 13D), resembling the shape of dendritic arbors in neuron #7 but with fewer ramifications (Figure 11A).

In summary, we identified a type of interneurons with sparse axonal branches; neuron #11 was a pyramidal neuron and neuron #12 had characteristic morphology, i.e., both dendrites and axons mostly spread over the deep layers of PreS. In both neurons, the dendritic and axonal domains directed toward distinct regions.

## Discussion

### Methodological Considerations

The present study used a single neuron-tracing method to demonstrate that each presubicular layer III neuron has various patterns of axonal collateralization. Our results indicate that layer III of PreS comprises several subtypes of intrinsic connection neurons and cortical connection neurons, including MEC- and/or Sub-projecting cells. The Sindbis virus vector used in the present study has already been demonstrated to infect neurons without preference with regard to specific neuronal type (Kuramoto et al., 2009), so our results appear useful for anatomical classification of neurons in layer III of PreS. This virus vector has been reported to not cause degenerative changes in neuronal processes over survival periods between 18 and 72 h (Furuta et al., 2001). In the present study, no obvious degenerative changes such as beading or disconnection in the axons were detected. We, therefore, speculate that our results demonstrate characteristics of the normal, not pathological, morphology of the axonal branches of presubicular layer III neurons. We have performed trials for determining the optimum conditions for virus infection and GFP expression; in particular, we have varied the volume, dilution and BSA concentration of injected virus solution, and also varied the survival time of infected animals. Accordingly, we could conceivably estimate optimal experimental conditions, which enabled us to visualize and reconstruct the previously unreported structures of single neurons, such as the intricate terminal arborization of single MEC projection neurons and axonal collateralization of single subicular and parasubicular projection neurons in layer III of PreS. In some cases, the distance between infected neurons was as short as 50 μm (such as neurons #6 and #7), but we could successfully trace axonal structures by identifying the cell of origin. In our results, several long, commissural and/or subcortical projection fibers could be found in some layer III neurons. One possibility is that other types and/or subtypes of layer III neurons may be identified by investigating commissural and subcortical projection patterns at a single neuronal level, for future studies.

### Band-Like Terminal Zones of MEC Projection Neurons

Our previous tracer study indicated that the presubicular projections terminated in the zone or band-like area in layer III of MEC perpendicular to MEC/LEC boundary and almost parallel with the rhinal fissure (Honda and Ishizuka, 2004). Here, we elucidated that the width (about 500 μm) and axis of these zones closely matched the terminal fields of the single neuronal projection shown in the present results (Figure 6). Using two-dimensional unfolded maps, we have previously demonstrated the topographic nature of presubiculo-entorhinal projections in that the septotemporal or longitudinal axis of PreS corresponds to the axis on MEC/LEC boundary. That is, the septal PreS projects to the band-like zone near the rhinal fissure and the temporal PreS projects to the band-like zone away from the rhinal fissure (Honda and Ishizuka, 2004). As seen in Figure 6, the patterns of entorhinal projection of both neurons #1 and #2 are consistent with a topographic manner in which the band-like terminal field of neuron #2 is located closer to ParS/MEC boundary than that of neuron #1, because the cell body of neuron #2 is located at a more temporal level than that of neuron #1. We speculate that both two presubicular layer III neurons are considered major types of entorhinal projection cells as previously reported for PreS HD cells (Preston-Ferrer et al., 2016). Multiple modules of grid cell clustering have recently been reported in rat MEC, with a band-like appearance dorsoventrally, but not mediolaterally (Stensola et al., 2012). Our findings suggest that single presubicular HD cells can simultaneously activate one or more band-like grid cell modules in the superficial layers of MEC and affect a wide range of grid-cell activities (Ray et al., 2017; Naumann et al., 2018). We have elucidated that the distribution pattern of cells with origins of CA1 and subicular projections was also arranged as band-like zones in layer III of EC in the rat and rabbit (Honda et al., 2012; Honda and Shibata, 2017). We, therefore, propose that such a band-like pattern in EC be inferred to be fundamental for memory function and highly conserved across animal species.

### MEC/Sub Projection Neurons

Similar to neurons #1 and #2, neuron #3 was a spiny pyramidal cell that projected to cortical areas other than PreS itself; all three, therefore, seem to represent excitatory neurons. Considering the long distance of axonal projection, neuron #4 is likely also excitatory, although the possibility that it represents a kind of long-range projection-type γ-aminobutyric acid (GABA)ergic neuron cannot be denied (van Haeften et al., 1997; Tomioka et al., 2005), given the relatively smooth dendritic arbors. Neurons #3 and #4 can also transmit signals to subcortical areas concurrently with the ipsilateral PreS, Sub and MEC, through axonal branches that enter into the fimbria/fornix, dorsal hippocampal commissure, or corpus callosum. The target areas remain uncertain because we cut-off both the corpus callosum and fimbria/fornix in the process of separating the hemispheres from the diencephalon in order to make serial sections perpendicular to the long axis of the hippocampal formation. Nonetheless, the commissural axonal branches from these neurons can presumably terminate in the contralateral MEC, as a major commissural projection of layer III in rat PreS (Honda and Ishizuka, 2004). As for the subcortical efferents, we propose the existence of several minor projections originating from layer III of PreS to the anterior thalamic nuclei and/or lateral mammillary nucleus, in addition to the major projections originating from deep layers of PreS (Huang et al., 2017; Simonnet and Fricker, 2018). In our results, all four MEC and MEC/Sub projection neurons gave off recurrent collaterals to layer VI of PreS. Accordingly, the targeted layer VI cells could receive the same excitatory signals as the other cortical or subcortical areas. Although the postsynaptic targets of these recurrent collaterals remain uncertain, several kinds of inhibitory neurons in layer VI of PreS could conceivably receive inputs and affect the microcircuit within PreS (Nassar et al., 2018). Moreover, synaptic connectivity among excitatory neurons reportedly increases in probability toward the deep layers of PreS in the rat (Peng et al., 2017). This suggests that excitatory signals from recurrent collaterals of MEC and/or MEC/Sub projection neurons can directly activate the subcortical projection neurons in layer VI.

### Subicular Projection Neurons

We have previously confirmed that in cases with wheat germ agglutinin-horseradish peroxidase injection into various parts of PreS, many cells labeled in a retrograde manner, but without any distinct deposits of anterograde labels could be detected in the pyramidal cell layer of Sub (Honda and Ishizuka, 2015). Accordingly, the subiculo-presubicular projection was mostly unidirectional, with few, if any, projections from PreS to Sub in the rat. Funahashi et al. (1999) elucidated presubicular inputs to Sub by slice electrophysiology, but the cells of origins were in the deep layers of PreS. The present results of a single neuron study revealed that layer III of PreS involved several kinds of subicular projection neurons, including neurons that innervate only Sub (such as neuron #5), unlike Sub/MEC projection neurons (neurons #3 and #4). Minor connections such as the presubiculo-subicular projection can be successfully detected by our labeling method. Axonal branches of all Sub and Sub/MEC projection neurons terminated in the pyramidal cell layer of Sub, but not in the molecular layer, suggesting that the proximal (near the cell body) position of dendritic or axonal arbors of the pyramidal neurons of Sub are more likely to receive inputs from layer III neurons of PreS.

### Parasubicular Projection Neurons

According to Nassar et al. (2015), some cluster 2 cells in mice that contained quasi-fast-spiking, basket-cell-like interneurons and were confirmed to be located at the distal part of PreS projecting into the nearby ParS and extensively ramifying within the superficial or deep layers of ParS. Our neuron #7 seemed to be a similar type because some of the axonal branches extended more distally toward ParS. Our results demonstrated that layer III of the distal PreS comprised several subtypes of neurons including parasubicular projection neurons such as neuron #6 and also interneurons such as neurons #7 and #12. Both interneurons tended to ramify more densely in the deeper layers and occupied regions distinct from the zones of dendrites. This suggests that presubiculo-parasubicular projection and its regulation by some kinds of intra- or interlaminar interactions can exist at least within the distal portion of PreS.

### Classification of Interneurons

From the perspective of morphological characteristics (regarded as interneurons with axons ramifying locally), five of our six intrinsic connection neurons in our results seem to be classifiable as inhibitory interneurons, while only neuron #11, a small, spiny pyramidal-type neuron, can be excitatory. Within the five presumable inhibitory interneurons, three (neurons #7, #9 and #10) showed densely spiny dendritic arbors. Some kinds of GABAergic interneurons in the rat neocortex are spiny (Kubota et al., 2011) and glutamatergic spiny pyramidal cells with locally confined axons that do not leave the cortical gray matter have also been reported (regarded as “intrinsic glutamatergic spiny cells” in DeFelipe et al., 2013). Rat GABAergic cortical interneurons have already been classified into many subtypes by their electrophysiological and morphological properties (Karube et al., 2004; Uematsu et al., 2008). We will need to evaluate the degree of axosomatic contacts, axon branching patterns, and bouton distribution quantitatively to collate reported data and our results. According to the classification of GABAergic interneurons by DeFelipe et al. (2013), most of our interneurons seem to be translaminar type, with axons not confined to a single layer. As shown in Figures 12, 13, our neurons #7 and #12 are both identified as displaced and descending types, because relative localizations of the dendritic and axonal arbors are shifted and not colocalized, and are depicted as mostly descending, radial arborizations. Neuron #8 seems to be identified as an intracolumnar type because the axonal arbors are confined to a single cortical column size at a diameter of 300 μm. On the other hand, neuron #9 can be defined as a transcolumnar type, because the axonal branches distributed for a distance >400 μm along the septotemporal axis. In the present study, our first priority was to visualize and completely trace the whole axonal morphology. We thus did not detect the expression of neuron markers such as parvalbumin and somatostatin. In mouse PreS, Simonnet et al. (2017) reported the existence of a pyramidal-Martinotti-cell feedback loop within layer III, which works as an inhibitory microcircuit for tuning and maintaining head direction signals in PreS. In the present study, we did not find Martinotti cells in layer III of rat PreS, but layer III pyramidal neurons in our results were also likely to participate in such feedback regulatory mechanisms. In another study using *in vitro* brain slices, Simonnet et al. (2013) classified the small regular spiking cells in layers II/III of rat PreS as “Cluster 1,” and also described that most cells in Cluster 1 are small, pyramid-shaped neurons. Some neurons in our results (including neurons #8 and #11) were similar to their Cluster 1 cells when only considering the dendritic morphology.

Superficial layers of rat MEC reportedly receive cell-type-specific intralaminar and ascending interlaminar feedback inputs, with those inputs attributed to the striking asymmetry of the deep to superficial microcircuitry within MEC (Beed et al., 2010). Moreover, Nilssen et al. (2018) reported that layer II of rat LEC contains “fan cells,” in which dendrites fan toward the direction of the pial surface with the axon descending to the deep layers, and that such fan cells were specifically connected to two types of inhibitory interneurons (fast spiking and non-fast spiking cells) in the vicinity of the cell body. Fan cells were suggested to be an important component of local circuits within the superficial layers of LEC. In the present study, neurons #11 and #12 could be considered as a kind of interneuron with a distinctive polarity in the dendritic and axonal domains and seem to participate in focal microcircuits, which straddle the neighboring cortical columns in rat PreS.

### Patterns of Distribution of Cortical and Intrinsic Projection Neurons

We divided layer III of PreS into multiple regions along the septotemporal, proximodistal and also superficial-to-deep axes, and demonstrated the locations of soma and terminations of axonal branches. In our study, as seen in Table 1, the majority of cortical (MEC/Sub, Sub, and ParS) projection neurons were distributed in regions from the septal to mid-PreS. Previously, the septal half of PreS (also called the postsubiculum) has been featured for its functional importance (Boccara et al., 2010; Bett et al., 2013) and our results raise the possibility that the septal half of PreS is much more divergent in neuronal subtypes, especially for cortical projections. On the other hand, no distinct distribution patterns that correlated with cell-type specificity could be found along proximodistal or superficial-to-deep axes. This may be partially due to an insufficient number of samples. We should collect more data for future studies, but it can be assumed that the larger the number of certain subtypes of neurons, the higher the infection probability, and if so, there is also a possibility that the proportion of each neuronal subtype in our study may reflect an actual quantitative statement to a certain extent, even with such a small total number (*n* = 12).

In conclusion, we have demonstrated the morphological features of the 12 layer III neurons of PreS, including cortical and intrinsic projection neurons, and have indicated the existence of multiple types of MEC projection neurons with distinct patterns of axonal collateralization. Layer III of PreS also comprises a wide variety of intrinsic projection neurons, which may play a critical role in mechanisms underlying head-directional signaling.

## Data Availability

The raw data supporting the conclusions of this manuscript will be made available by the authors, without undue reservation, to any qualified researcher.

## Ethics Statement

The present experiments were approved by the Animal Care and Use Committee of Tokyo Women’s Medical University, and all conformed to the Guidelines for the Care and Use of Laboratory Animals (National Institutes of Health, Bethesda, MD, USA). We used eight adult male Wistar rats (280–305 g body weight; Clea Japan, Tokyo, Japan), and every effort was made to minimize the number of animals used and the pain and distress of animals.

## Author Contributions

All authors had full access to all the data in the study and take responsibility for the integrity of the data and the accuracy of the data analysis. YH and TF: study concept, design, drafting of the manuscript, analysis and interpretation of data. YH: acquisition of data and obtained funding. TF: critical revision of the manuscript for important intellectual content.

## Conflict of Interest Statement

The authors declare that the research was conducted in the absence of any commercial or financial relationships that could be construed as a potential conflict of interest.

## Abbreviations

AB: angular bundle
CA: cornu ammonis
cc: corpus callosum
dhc: dorsal hippocampal commissure
dist: distal
EC: entorhinal cortex
GFP: green fluorescent protein
hf: hippocampal fissure
ld: lamina dissecans
LEC: lateral entorhinal cortex
MEC: medial entorhinal cortex
mol: molecular layer
ParS: parasubiculum
pcl: pyramidal cell layer
PreS: presubiculum
prox: proximal
pyr: pyramidal
rf: rhinal fissure
RS: retrosplenial cortex
sept: septal
Sub: subiculum
temp: temporal.
